# Coronal alignment-related mediolateral load distribution and polyethylene wear in a medial-pivot total knee arthroplasty design

**DOI:** 10.1007/s00402-026-06558-z

**Published:** 2026-10-30

**Authors:** Celina Lesnicar, Manuel Kistler, Christoph Thorwächter, Irem Tural, Johanna-Maria Simon, Thomas Richard Niethammer, Boris Michael Holzapfel, Matthias Woiczinski

**Affiliations:** 1 Department of Orthopaedics and Trauma Surgery, Musculoskeletal University Center Munich (MUM), Ludwig-Maximilians-Universität München, Munich, Germany; 2 Experimental Orthopaedics University Hospital Jena, Waldkliniken Eisenberg, Friedrich Schiller University Jena, Jena, Germany

**Keywords:** Wear, Varus, Valgus, KAM, TKA

## Abstract

**Introduction:**

While mechanical alignment in total knee arthroplasty aims for a neutral hip-knee-ankle angle, kinematic alignment preserves the native joint line, often allowing residual varus or valgus. Clinically, constitutional variations in alignment affect the coronal joint moments and the mediolateral load distribution, but the impact on polyethylene wear remains unclear.

**Materials and methods:**

Wear of a medial pivot total knee arthroplasty insert (MS 5C^®^, implantcast GmbH, Germany) was evaluated in a knee wear simulator replicating the walking cycle. Inserts were tested under three conditions representing overall lower-limb coronal alignment and the associated mediolateral load distribution: neutral (60% medial / 40% lateral), varus (70% / 30%), and valgus (50% / 50%), with three (*n* = 3) dynamically loaded inserts and one load-soak control per condition. Testing followed ISO 14243-1:2009 over 5 million cycles. Gravimetric wear and wear patterns were assessed.

**Results:**

A numerical trend was observed among the groups: The varus-related load distribution demonstrated the lowest overall wear (4.83 ± 1.06 mg/million cycles), whereas the valgus group produced the highest wear (9.43 ± 1.47 mg/million cycles); the neutral group was intermediate (6.81 ± 3.07 mg/million cycles). However, these differences did not reach statistical significance. Visual inspection revealed medial-dominant wear in all groups, with increased lateral wear under valgus-related load distribution.

**Conclusions:**

Overall lower-limb coronal alignment and the associated mediolateral load distribution appear to influence polyethylene wear in total knee arthroplasty. The observed numerical trend suggests that alignment-related changes in mediolateral load distribution may influence polyethylene wear patterns in this medial-pivot TKA design. Further studies are required to determine the clinical relevance of these observations.

## Introduction

Traditionally, most surgeons have aimed for a neutral mechanical alignment (MA), with the goal of restoring a hip-knee-ankle (HKA) angle within ± 3° of neutral to optimize load distribution and implant survival [1, 2]. More recently, the field has moved toward more individualized alignment strategies that do not enforce a fixed neutral target, including kinematic alignment (KA), which aims to restore the patient’s native anatomy and joint line, and functional alignment (FA), which adjusts component positioning based on patient-specific soft-tissue balance and gap balancing [3, 4]. These concepts are increasingly guided by phenotypic classification systems, such as the functional knee phenotype classification [5] and the Coronal Plane Alignment of the Knee (CPAK) classification [6], which categorize knees according to their constitutional coronal alignment and joint line obliquity. A central feature of these individualized approaches is the deliberate acceptance of physiological leg axis deviations [5, 6]. Several studies have reported that KA provides improved functional outcomes, a more physiological perception of the knee, and gait patterns closer to the natural knee, while implant survival and complication rates remain comparable to MA [7, 8]. However, the long-term biomechanical implications of KA, particularly with regard to polyethylene (PE) wear, remain limited [9–11].

Deviations in coronal alignment directly affect how loads are distributed across the tibiofemoral joint during gait, which in turn influences mechanical stress on the PE insert and may affect its long-term wear behavior [11]. This can be quantified via the knee adduction moment (KAM), the external frontal-plane moment generated by ground reaction forces during gait, which reflects the relative medial/lateral load share [12]. Under physiological conditions, approximately 60 % of the load is borne medially and 40 % laterally. Overall lower-limb varus alignment increases the medial share to around 70 % and decreases the lateral share to 30 %, while valgus alignment results in a more balanced distribution, approximately 50 % / 50 % [13]. Clinically, residual (unintended) varus alignment after MA-total knee arthroplasty (TKA) increases KAM and elevates medial compartment stress [14], whereas KA-TKA has been shown to reduce KAM compared with MA-TKA, even when varus alignment is maintained, likely due to a more floor-parallel tibial joint line reducing the effective moment arm of the ground reaction force [15]. These gait-based findings are supported by structural and experimental evidence:

Retrieval analyses of posterior stabilized (PS) and cruciate-substituting (CS) knee designs have shown that significantly greater PE surface damage, including scratching and pitting, was exhibited in knees that maintained (unintended) varus alignment after surgery [10]. Simulator and cadaveric studies demonstrated that even small deviations of 1° − 3° in coronal alignment can alter contact stresses and load transmission across the tibial PE surface, whereas investigations indicate that varus alignment exceeding 3° markedly increases tibial insert strain and accelerates wear [11, 16, 17].

As KA principles and individual coronal alignment targets become more widely adopted, further insights into how altered mediolateral load distributions influence in vitro wear patterns are essential to optimize contemporary TKA designs for these individual kinematic concepts.

The aim of the present study was therefore to investigate the effect of three alignment-related mediolateral load distributions representing neutral (60 % medial / 40 % lateral), varus (70 % / 30 %), and valgus (50 % / 50 %) overall lower-limb alignment on the PE wear performance of a modern bicondylar TKA in a standardized simulator environment. We hypothesized that the varus- and valgus-related load distributions would result in higher wear rates compared to the neutral condition.

## Materials and methods

### Implant and embedding

For the in vitro wear analysis, a total of twelve (n = 12) medial pivot total knee implants (MS 5C^®^ Knee System, implantcast GmbH, Buxtehude, Germany) with a single-radius femoral component, and a fixed-bearing insert with medial congruency and a flat lateral compartment were used. All components corresponded to size 7L. The femoral components (5C^®^ MEDic Knee System) and the tibial trays (5C^®^ Knee System) were manufactured from cobalt-chromium-molybdenum alloy in accordance with ISO 5832-4. The tibial inserts (5C^®^ MEDic Knee System) consisted of ultra-high molecular weight PE (UHMW-PE; GUR 1020; γ-sterilized ≥ 25 kGy and packed under inert gas) with vitamin E stabilization (implacross^®^ E) and a size specification of 9 mm.

**Fig. 1:**
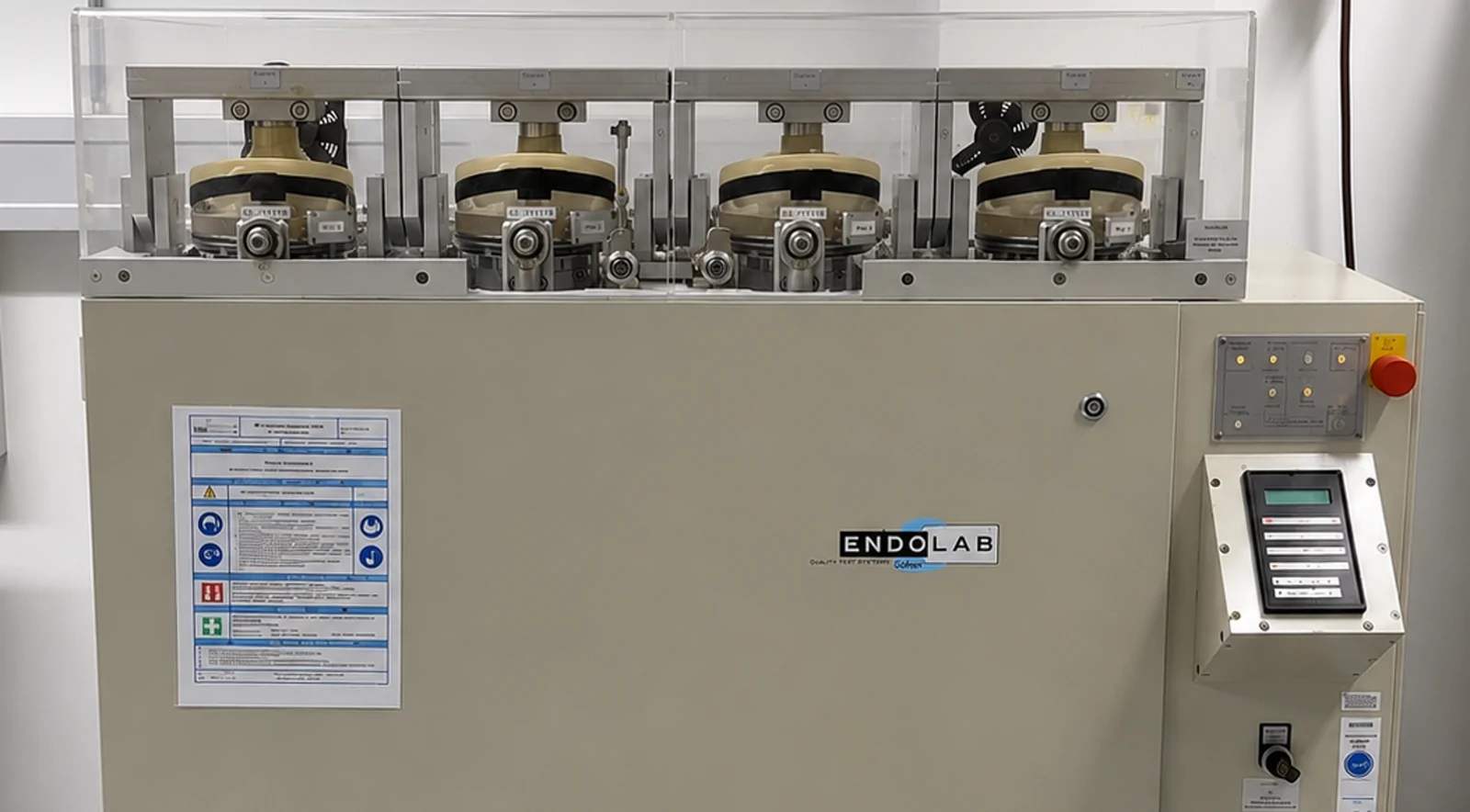
Experimental setup of the four-station servo-hydraulic knee wear simulator (EndoLab GmbH, Thansau, Germany), including the femoral and tibial fixation system used for wear testing according to ISO 14243-1:2009. From left to right: one load-soak control (station 0) and three dynamically loaded total knee arthroplasty implants (stations 1 – 3)

The femoral components were embedded in polyurethane resin (RenCast^®^ FC 52/53) and mounted onto the simulator using a stainless-steel tube. The tibial components were likewise embedded in polyurethane resin and secured via a tibial fixation system (Fig. 1). This embedding and fixation approach follows ISO 14243-1 standards and has been used in previous simulator studies [9, 18, 19].

**Fig. 2:**
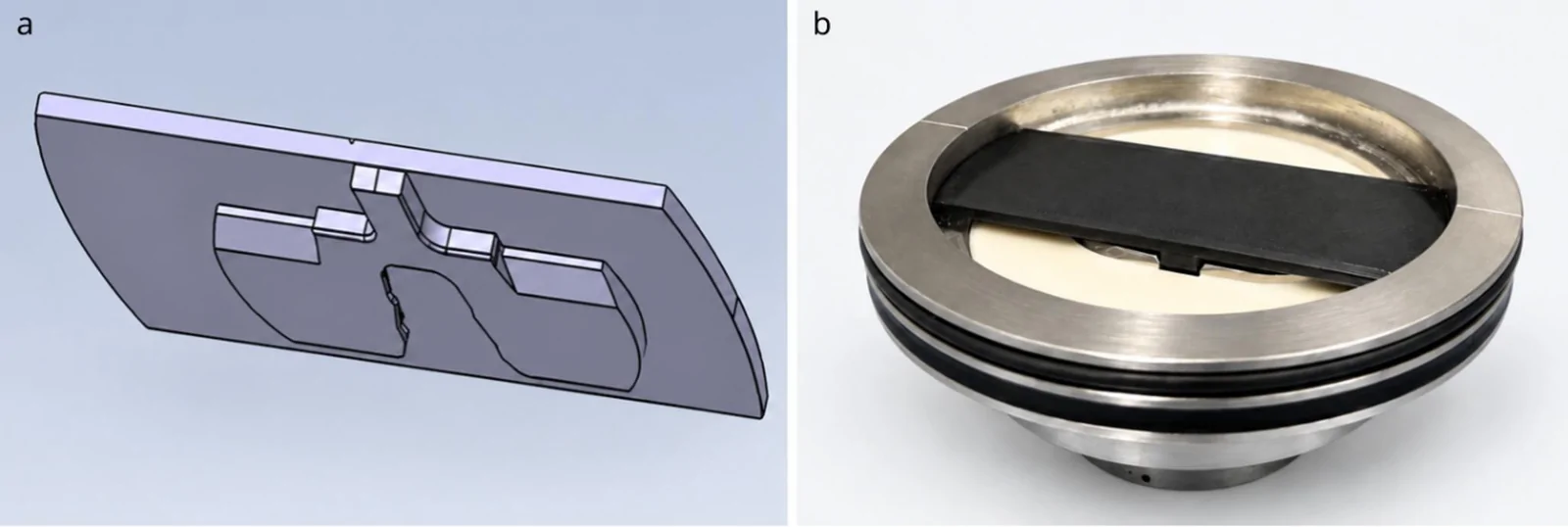
Custom 3D-printed positioning guide used for specimen embedding, shown exemplarily for the neutral condition. **a** Bottom isometric view of the CAD representation of the guide, illustrating the outer support geometry and the insert-shaped central holder used to clip and position the tibial tray. **b** Application of the guide during specimen preparation within the embedding container. Corresponding guides for the varus and valgus loading conditions were identical in outer shape but featured a mediolaterally shifted central holder

To replicate the mediolateral load distributions associated with neutral, varus, and valgus overall lower-limb alignment, three custom 3D-printed positioning guides were designed in CATIA V5 (Dassault Systèmes, Vélizy-Villacoublay, France), with one guide corresponding to each loading condition. The guides were shaped similarly to a tibial insert and clipped into the tibial tray during embedding. Their outer geometry rested on the inner rim of the embedding container, while the insert-shaped central holder, into which the tibial tray was clipped, was positioned at a predefined condition-specific mediolateral position for the neutral condition and shifted medially or laterally for the varus and valgus conditions, respectively. In this way, the tibial tray was maintained at a predefined mediolateral position relative to the embedding container without any coronal rotation. The magnitude of the required mediolateral offset was calculated according to ISO 7207-1 based on the width (*w*) of the tibial component using the relationship Offset_medial_ = 0.07 *w* ± 0.01 *w*. After filling the embedding container with polyurethane resin, the positioning guide was removed once the tibial component had been fixed in the predefined position. Figure 2 shows the neutral positioning guide as an example of the guide design and its use during specimen preparation.

**Fig. 3:**
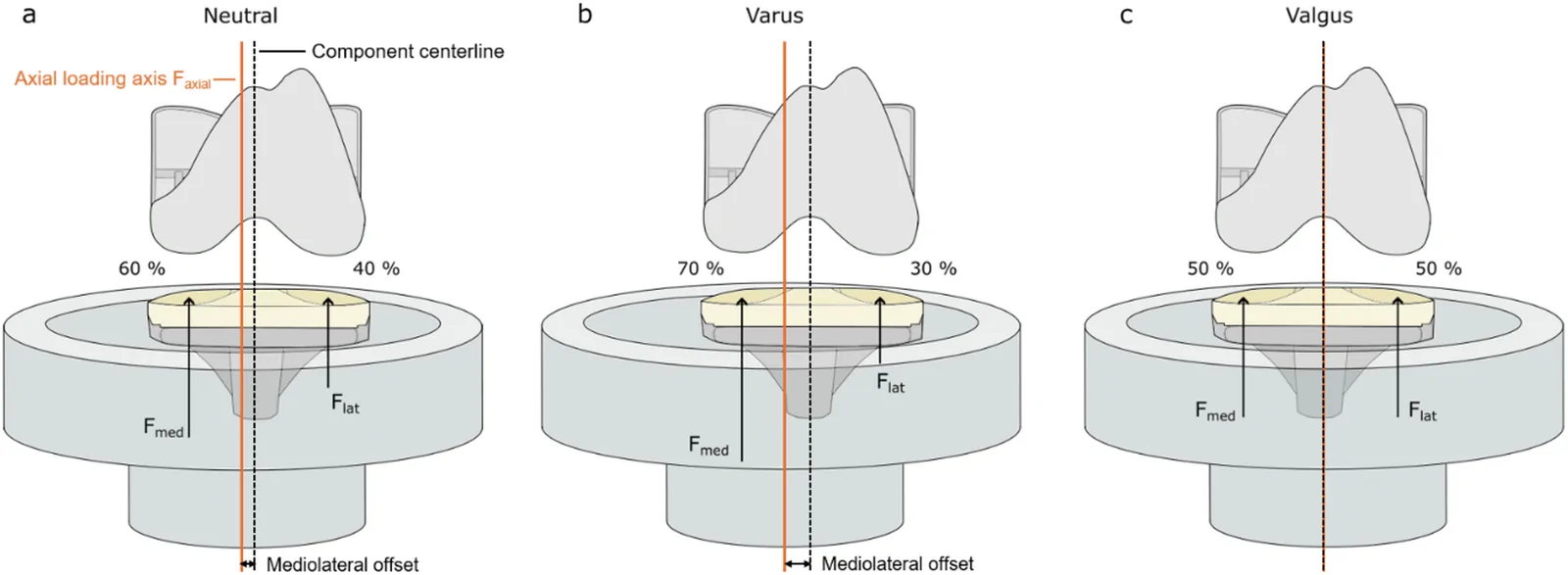
Schematic illustration of the three experimental loading configurations representing **a** neutral, **b** varus, and **c** valgus overall lower-limb alignment, with corresponding medial (F_med_) and lateral (F_lat_) load distributions generated by varying the mediolateral offset between the unchanged axial loading axis (F_axial_) and the component centerline. The solid orange line indicates the axial loading axis (F_axial_), whereas the black dashed line represents the component centerline

This approach resulted in three testing groups, consisting of four samples each, representing the mediolateral load distributions associated with equivalent neutral (0°, 60 % medial/ 40 % lateral), 3° varus (70 % / 30 %), and 3° valgus (50 % / 50 %) overall lower-limb mechanical axis conditions. The mediolateral load distributions were adapted based on the study by Halder et al. (2012) [13]. The stated 3° varus and valgus conditions therefore refer to equivalent overall lower-limb mechanical axis conditions associated with these load distributions rather than actual component angulation. The position and orientation of all femoral components at mounting remained unchanged across the three loading conditions. Illustrative representations of the three experimental loading configurations are shown in Fig. 3.

### Wear simulator testing

**Fig. 4:**
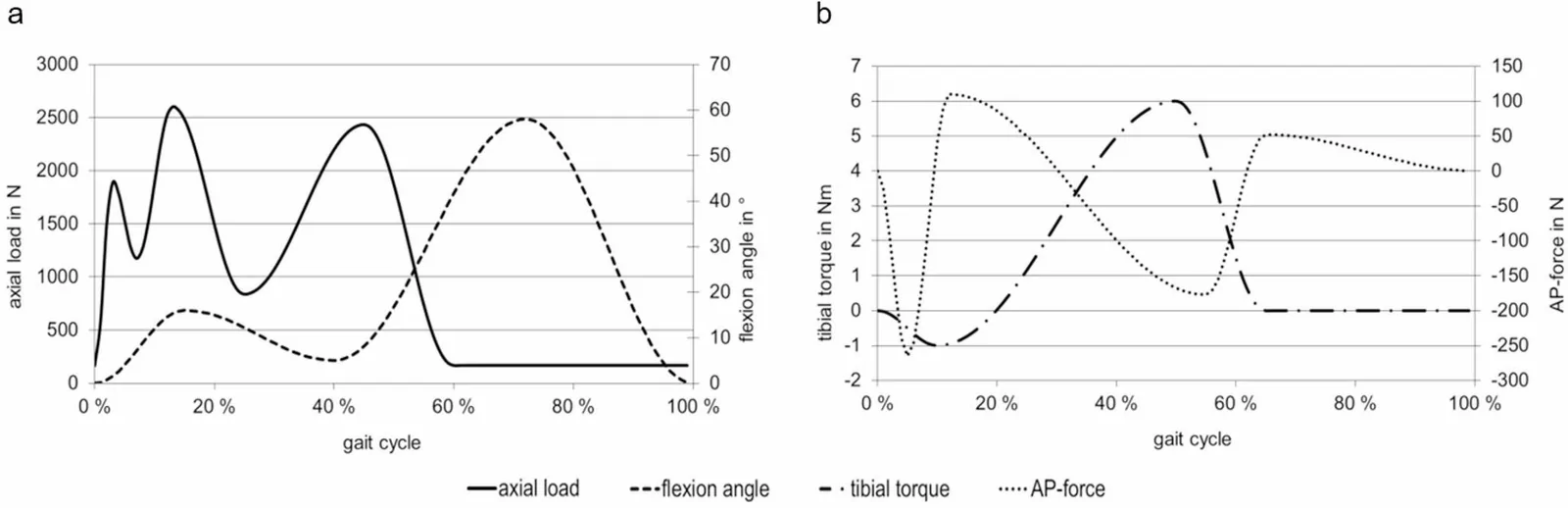
Loading and motion profiles applied by the servo hydraulic knee wear simulator according to ISO 14243-1:2009: **a** axial loads and flexion angles and **b** tibial torques and AP-forces during one load cycle

Wear testing was conducted according to the ISO 14243-1:2009 protocol, employing a customized servo hydraulic knee wear simulator (EndoLab GmbH, Thansau, Germany). The simulator continuously recorded anteroposterior (AP) translation and internal-external (IE) tibial rotation as feedback signals for simulator control and to monitor compliance with the prescribed ISO 14243-1:2009 protocol. For each loading configuration, four implants were tested over 5.0 million cycles, simulating level walking as defined by ISO 14243-1:2009 (Fig. 4). In each test group (neutral, varus, valgus), three implants (1 – 3) were dynamically loaded, while one additional TKA specimen (0) served as a load-soak control, only experiencing axial compression without flexion, AP-force or tibial torque.

All wear tests were carried out in a lubricated environment to replicate physiological joint conditions. A mixture of newborn calf serum (Biochrom, Berlin, Germany) and distilled water was used to achieve a protein concentration of 20 g/l. Each testing chamber was filled with 300 ml of this solution, ensuring that all implant components remained fully submerged throughout the test. To maintain the stability of the medium, 24 g/l EDTA (AppliChem, Darmstadt, Germany) was added to buffer the pH, and 10 ml/l Amphotericin B (Biochrom, Berlin, Germany) was used to prevent fungal contamination. An elastic membrane was used to seal the test chambers, preventing contamination as well as maintaining a constant temperature of 37 °C throughout the experiments, thereby ensuring physiologically relevant conditions.

Prior to testing, all UHMW-PE inserts were conditioned in the lubricant solution. The inserts were repeatedly weighed using an analytical balance (Sartorius BP211D, Germany) with a precision of 0.01 mg, following the gravimetric procedure defined in ISO 14243-2:2016. Conditioning was considered complete, once the incremental mass change over a 24-hour period was less than 10 % of the previous cumulative mass gain, indicating saturation. Gravimetric wear was later corrected for both load soak controls and air buoyancy to ensure accurate mass loss determination.

### Weight analysis

The lubricant, including all additives (EDTA, Amphotericin B), was replaced every 0.5 million cycles. After 0.5 million cycles, and subsequently at 1.0 million cycle intervals, the UHMW-PE inserts were gently cleaned and weighed in accordance with the ISO 14243-2:2016. Wear was calculated as mass loss relative to the initial mass and corrected for both load-soak controls and air buoyancy. Wear rate was expressed as mg/million cycles.

### Wear pattern analysis

Additionally, the inserts were scanned at each of the intervals mentioned above (Epson Expression 1680 Pro, Seiko Epson, Tokyo, Japan) at 300 dpi with a scaling of 1 mm. Wear patterns were qualitatively evaluated based on the serial scans. The primary assessment was performed by one evaluator and reviewed by a second evaluator blinded to the loading condition. Discrepancies were discussed and resolved by consensus. No formal inter-observer reproducibility analysis was performed. Photographic documentation of all metallic components (femoral and tibial) was performed before and after the wear simulation to detect potential surface damage such as scratches or abrasion. Images were taken using a Nikon D5500 digital camera equipped with a Nikon DX 18–105 mm lens and a + 5-diopter macro attachment.

### Data analysis and statistics

Individual wear rates were determined as the slopes of linear regressions of gravimetric wear against cycle count and are reported with the corresponding standard error of the slope. Group wear rates are reported as mean ± standard deviation (SD) across the three dynamically loaded specimens per condition. The study was designed as an exploratory in vitro wear simulation using three dynamically loaded specimens and one load-soak control per condition, corresponding to the four-station simulator configuration. No a priori sample size calculation was performed. Given the analyzed sample size n = 3 dynamically loaded implants per group), normality tests were not applicable. Differences in wear rates between the three loading conditions (neutral, varus, valgus) were assessed using the non-parametric Kruskal-Wallis test, followed by Dunn’s multiple comparisons test for post-hoc pairwise analyses. To complement the hypothesis test, the effect size for the Kruskal-Wallis analysis was expressed as epsilon-squared (ε²). Statistical significance was considered at *p* < 0.05. The relationship between gravimetric wear and cycle count was evaluated using linear regression. The goodness of fit was expressed as the coefficient of determination (R²), with values above 0.7 considered indicative of a strong fit, values between 0.3 and 0.7 of a moderate fit, and values below 0.3 of a weak or negligible fit. All analyses were performed using GraphPad Prism (version 10.2.3, GraphPad Software, San Diego, CA, USA).

## Results

### Wear rate

All dynamically loaded specimens completed testing over 5.0 million cycles. No specimens or wear measurements were excluded from the analysis, and no technical problems occurred that affected the validity of the experimental data. The mean wear rate was 6.81 ± 3.07 mg/million cycles in the neutral group, 4.83 ± 1.06 mg/million cycles in the varus group, and 9.43 ± 1.47 mg/million cycles in the valgus group. The corresponding cumulative gravimetric wear after 5.0 million cycles was 36.32 ± 16.83 mg, 23.60 ± 5.68 mg, and 48.40 ± 7.46 mg, respectively. Statistical analysis revealed no significant differences between the three loading conditions (Kruskal-Wallis test, H(2) = 4.36, *p* = 0.113, ε² = 0.39). Post-hoc Dunn’s multiple comparisons confirmed that none of the pairwise differences reached statistical significance. Accordingly, the observed differences between groups represent numerical trends rather than statistically demonstrated differences. All three groups demonstrated a correlation between gravimetric wear and cycle count. Detailed values for individual specimens and the regression data are provided in Table 1.

**Table 1:** Wear data after 5.0 million cycles for the three dynamically loaded inserts (1 – 3) under the neutral, varus, and valgus loading conditions, corrected for load-soak effects and air buoyancy

Specimen	Gravimetric wear in mg			Wear rate in mg/million cycles			Goodness of linear regression (R²)		
	Neutral	Varus	Valgus	Neutral	Varus	Valgus	Neutral	Varus	Valgus
1	40.80	25.35	43.63	7.00 ± 0.51	5.25 ± 0.35	8.77 ± 1.07	0.98	0.98	0.95
2	50.46	17.25	57.00	9.78 ± 1.13	3.62 ± 0.34	11.12 ± 0.85	0.95	0.97	0.98
3	17.71	28.21	44.58	3.64 ± 0.61	5.62 ± 0.27	8.41 ± 0.50	0.90	0.99	0.99
Mean ± SD	36.32 ± 16.83	23.60 ± 5.68	48.40 ± 7.46	6.81 ± 3.07	4.83 ± 1.06	9.43 ± 1.47	0.94 ± 0.04	0.98 ± 0.01	0.97 ± 0.02

Individual wear rates are reported as regression slope ± standard error of the slope; group wear rates are reported as mean ± SD across the three dynamically loaded specimens

### Visual investigation of wear patterns

#### Topside wear

**Fig. 5:**
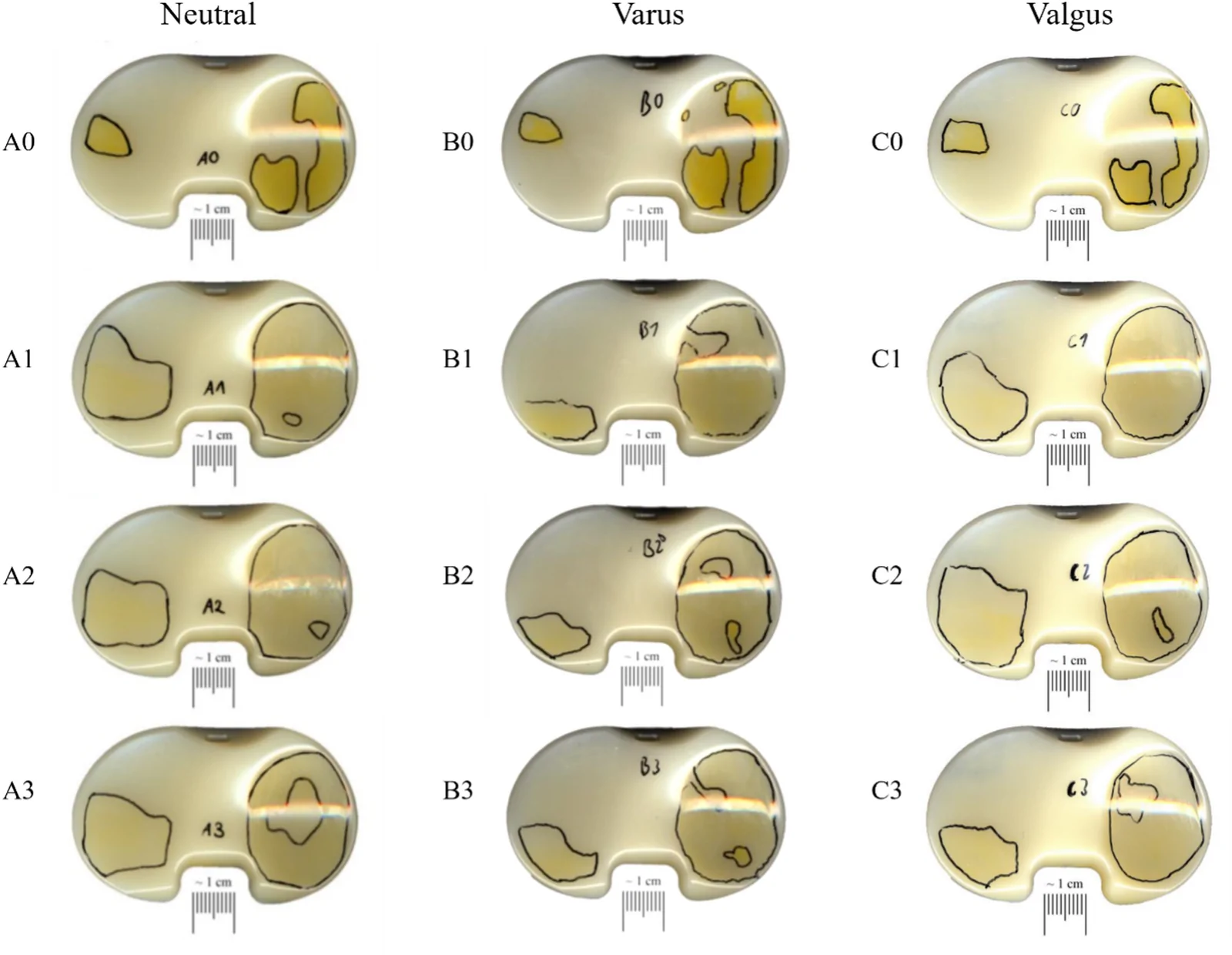
Wear traces (encircled areas) after 5.0 million cycles on the topside of the UHMW-PE inserts of the neutral (**A**), varus (**B**) and valgus (**C**) groups (left knee); insert 0: load-soak control with axial compression, inserts 1 – 3: full simulator loading

After 5.0 million cycles, all UHMW-PE inserts from the three loading conditions (neutral, varus, valgus) exhibited visible wear tracks on the articular surface (Fig. 5). Small surface scratches occurred sporadically. No indications of delamination or pitting were detected. Burnishing was observed on all inserts across all groups and was predominantly located on the medial compartment. Qualitative visual assessment indicated the smallest overall visible wear areas on the articular surface in the varus group. The neutral and valgus groups showed more extensive lateral burnishing than the varus group.

#### Backside wear

**Fig. 6:**
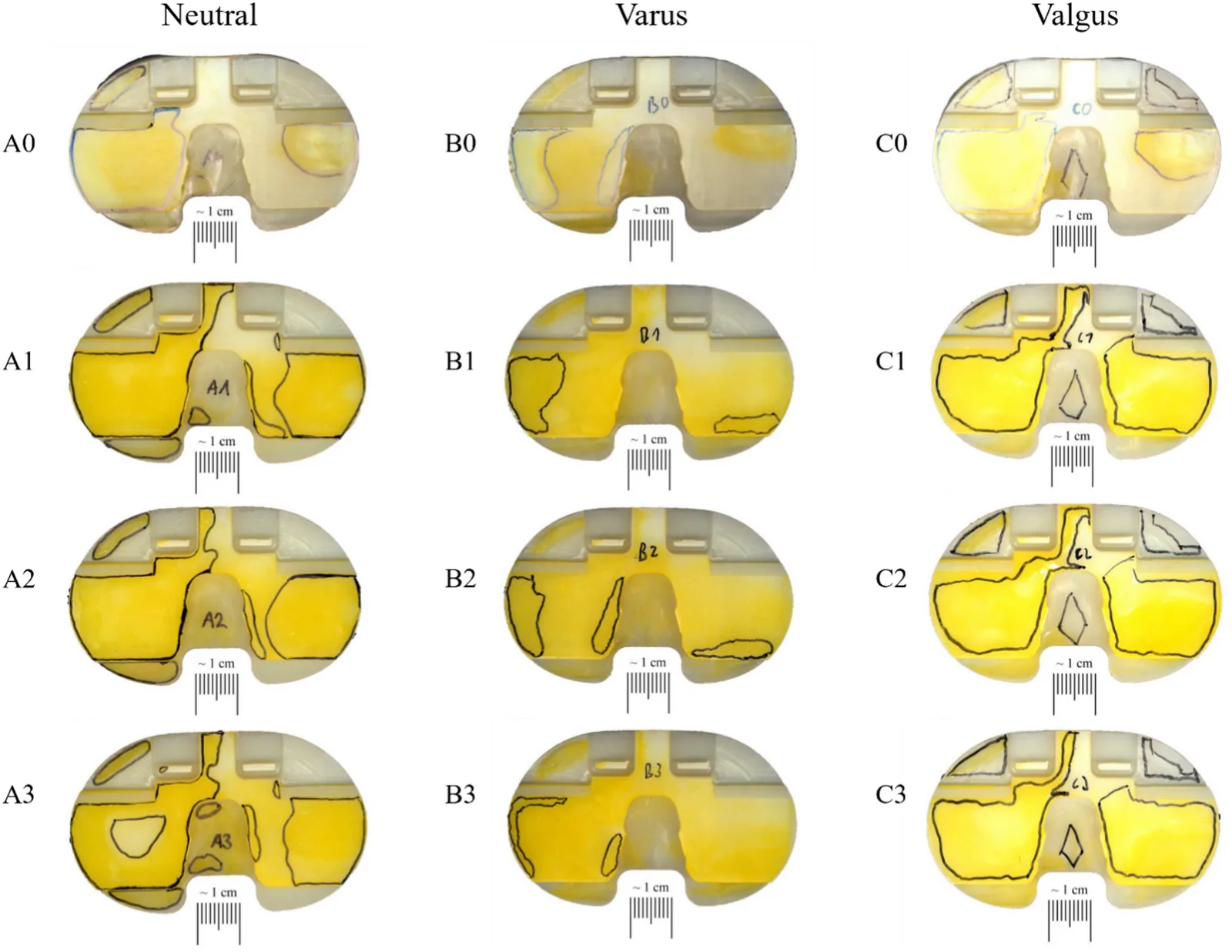
Wear traces (encircled areas) after 5.0 million cycles on the backside of the UHMW-PE inserts of the neutral (**A**), varus (**B**) and valgus (**C**) groups (left knee); insert 0: load-soak control with axial compression, inserts 1 – 3: full simulator loading

Burnishing and fine scratches were observed on the backside surfaces of all UHMW-PE inserts across the three groups after 5.0 million cycles (Fig. 6). No delamination or pitting was detected in any specimen. Qualitative visual assessment indicated the smallest backside wear areas in the varus group and the largest in the valgus group, with more extensive wear observed laterally in the valgus condition.

Regarding the metal surface of the tibial and femoral components, small, narrow scratches were observed after 5.0 million cycles.

## Discussion

This study revealed numerical trends in the magnitude and compartmental pattern of PE wear across the three alignment-related mediolateral load distributions (neutral, varus, valgus) in a modern medial pivot TKA design, without statistically significant differences in overall wear rate.

### Wear rate

The original hypothesis that both the varus- and valgus-related load distributions would result in higher wear than the neutral condition was not supported. The observed wear rates (neutral: 6.81 ± 3.07 mg/million cycles; varus: 4.83 ± 1.06 mg/million cycles; valgus: 9.43 ± 1.47 mg/million cycles) fall within the range reported for force-controlled knee wear simulator studies using conventional and highly cross-linked UHMW-PE, which spans approximately 3 – 40 mg/million cycles for cruciate-retaining (CR) TKA and about 2 – 33 mg/million cycles for PS TKA designs [20]. Wear rates toward the lower end of this range are typically reported for highly cross-linked PE, while conventional, non-cross-linked inserts tend to produce wear rates at the upper end [20]. The values in this study are therefore consistent with the expected performance of vitamin E-stabilized, cross-linked UHMW-PE.

Although no statistically significant differences in wear rates were observed among the three loading conditions, the numerical trend toward the lowest overall wear under the varus-related load distribution parallels the result of comparable studies using a similar experimental design, where 5° of varus or valgus tibial component positioning in a medial fixed-bearing unicompartmental design significantly reduced wear compared with neutral alignment (neutral: 12.16 ± 1.26 mg/million cycles; varus: 6.30 ± 1.38 mg/million cycles; valgus: 4.96 ± 2.47 mg/million cycles), attributing this to a reduced articulation contact area under the altered alignment condition [21]. These findings are consistent with earlier cadaveric and simulator work: Werner et al. demonstrated that pressure distribution and total load across the medial and lateral tibial changed significantly with as little as 3° of coronal component angulation, both during simulated trial reduction and knee simulator testing [11]. Similarly, in a mobile-bearing simulator study testing 1° to 3° alteration of tibial component alignment, Löer and Plitz concluded that deviations up to 3° remained within an acceptable range of in vitro tolerability, whereas larger deviations produced unacceptable loading conditions in their simulator setup [16]. Taken together, these studies support 3° as an approximate threshold beyond which coronal deviations begin to meaningfully alter tibiofemoral load transmission – consistent with the magnitude of overall lower-limb alignment represented by the loading conditions in the present study. Schroeder et al. reported comparable wear rates between kinematically and mechanically aligned components using a CR design [9]. In their setup, the femoral component was tilted by 4° relative to the tibial resection plane to simulate the joint-line obliquity typical of KA, while the overall mechanical loading axis of the simulator was kept unchanged. Consequently, the KAM itself was not directly altered, and their model isolates the effect of component-level joint-line tilt rather than of a shifted mediolateral load distribution. Despite this methodological difference, they too observed a trend toward reduced wear under the tilted (non-neutral) alignment condition, relative to neutral alignment [9].

### Visual investigation

Qualitative visual assessment indicated greater medial than lateral wear across all groups, reflecting the physiological predominance of medial loading during gait; in vivo instrumented-implant data confirm that the medial compartment typically transmits a greater share of the tibiofemoral contact force than the lateral compartment across the majority of the gait cycle, largely independent of overall limb alignment [13]. This is consistent with the medial-dominant loading pattern assumed even in the neutral condition (60 % /40 %).

One possible biomechanical explanation for this observation is the specific geometry of the tested medial-pivot design. The highly congruent, ball-in-socket medial compartment distributes load over a large contact area with correspondingly low contact stress, while the comparatively flat lateral tibial surface permits greater AP and rotational sliding of the femoral condyle [22]. This distinction matters because PE wear is driven disproportionately by cross-shear motion (i.e., sliding transverse to the predominant direction of motion) rather than by contact pressure alone [23]. Under the valgus-related load distribution, the increased lateral load therefore acts on the compartment that is both less congruent and potentially subject to greater cross-shear motion, which may explain the more extensive visually observed wear in this group. Under the varus-related load distribution, by contrast, load is concentrated on the more congruent, lower-shear medial compartment, while the lateral compartment is relatively offloaded, consistent with the qualitatively smaller visible wear areas observed in this group despite the higher local medial load. The neutral and valgus-related load distributions, in comparison, distribute load more evenly across both compartments, allowing wear to accumulate on both the high-shear lateral and the high-congruence medial surface, which may contribute to the numerically higher total material loss observed in these conditions.

This pattern is consistent with Schroeder et al., who similarly found that altered alignment conditions did not necessarily increase total wear but rather redistributed it between compartments [9]. However, Li et al. showed that residual varus alignment can still lead to progressive PE damage over time in vivo, likely reflecting the persistence of an elevated knee adduction moment even after surgical correction [10].

### Limitations

Some limitations should be considered when interpreting these results. The study was performed under in vitro conditions, which inherently limit the transferability to the clinical situation. In vivo, the knee joint is surrounded by ligaments, muscles, and other soft tissues that influence joint kinematics and load distribution. However, standardized knee wear simulation according to ISO 14243-1:2009 allows highly controlled and reproducible assessment of PE wear, providing valuable comparative data between different loading conditions. Furthermore, the small number of dynamically loaded specimens (n = 3 per condition) limits the statistical power of the study and may have prevented the detection of differences between loading conditions. Therefore, the absence of statistical significance should not be interpreted as evidence of equivalence between the groups. Level walking according to ISO 14243-1:2009 was simulated, which has been shown to account for the majority of the cumulative loading cycles experienced by the knee joint in daily life, making it the most relevant activity for assessing long-term wear [24, 25]. Higher load activities such as stair climbing or sports could reveal additional wear patterns and risks. Further, the findings are specific to the implant design tested and may not directly translate to other geometries. Nevertheless, medial pivot geometries are the design most commonly paired with KA and FA concepts in current clinical practice, supporting the clinical relevance of this combination to the research question [26]. This study was explicitly designed to isolate the effect of mediolateral load distribution by testing three predefined, controlled loading conditions representing neutral, varus, and valgus overall lower-limb alignment. Accordingly, the experimental conditions do not reproduce the full complexity of clinical KA or FA, in which component positioning, joint-line orientation, soft-tissue balance, and gap balancing may vary simultaneously. Therefore, the present findings should be interpreted as reflecting the isolated effect of mediolateral load distribution rather than the overall effect of a specific clinical alignment strategy. The accuracy and reproducibility of the custom positioning guides were not quantitatively validated by measuring the deviation between the predefined and achieved mediolateral position, which represents an additional methodological limitation. Other combinations of tibial and femoral component positioning – such as femoral component rotation, which also differs between KA and MA concepts – were deliberately kept constant to avoid confounding factors. In vivo, such additional variations in component positioning could further influence load distribution and potentially exacerbate PE wear beyond what was modeled in this study.

## Conclusions

The valgus-related load distribution showed a numerical trend toward the highest overall wear, whereas the varus-related load distribution showed the lowest total material loss in the tested medial-pivot implant design. However, no statistically significant differences in wear rate were detected between the three loading conditions. These numerical trends suggest that isolated coronal alignment-related changes in mediolateral load distribution may influence the distribution of PE wear and, to a lesser extent, the magnitude of PE wear in TKA. Furthermore, this effect may interplay with the specific geometry of the tibial insert. The observed numerical trend toward higher wear under the valgus-related load distribution may warrant further investigation, particularly in implant designs with a flat lateral tibial surface. Conversely, the varus-related load distribution demonstrated a numerically lower wear rate than the neutral and valgus conditions in this specific medial-pivot design. Further studies with larger sample sizes, different implant designs and more comprehensive models of clinical alignment strategies are required to determine the clinical relevance of these observations.

## Data Availability

The data that support the findings of this study are available from the corresponding author upon reasonable request.
